# In this month’s Bulletin 

**DOI:** 10.2471/BLT.26.000226

**Published:** 2026-02-01

In the editorial section, Viroj Tangcharoensathien et al. (62) introduce this theme issue to accompany the Prince Mahidol Award conference on demographic change, summarizing authors’ descriptions of relevant multisectoral policy and health systems responses. Jagadish K Chhetri et al. (63) point to improved long-term care provision in the context of population ageing. Jintana Jankhotkaew et al. (64) call for papers on commercial determinants of health. 

Gary Humphreys (67–68) reports on how an innovative model of integrated care is helping countries respond to the challenges of population ageing. Priscilla Idele speaks to Humphreys (69–70) about fertility, choice, and changing norms from the perspective of the data and analytics branch at the United Nations Population Fund. 

## China 

### Community support for people with dementia

Qiuling An and Fei Sun (112–117) list programmes in Shanghai.

## Thailand 

### Treating HIV, viral hepatitis and sexually transmitted infections

Tanyaporn Wansom et al. (71–81) analyse outcomes of integrated care provision.

### Effects of filial piety 

Nopphol Witvorapong et al. (131–133) consider how caregiving for older parents influences reproductive choices.

## WHO African Region 

### Medicines for older people 

Ke Wei Foong et al. (82–93) chart contents of guidelines and essential medicines lists. 

## WHO European Region

### Prevention of early mortality

Stuart Gietel-Basten et al. (94–102) weigh pronatalist policies against investments in health. 

## Global

### Adaptation of health information systems 

Ana Mendez-Lopez et al. (103–111) list changes needed to support age-friendly policies and services.

### Tests for a novel respiratory pathogen

Claudia M Denkinger et al. (118–120) present a diagnostic protocol for the first few cases.

### Medically-assisted reproduction

Gitau Mburu et al. (121–123) detail requirements for infertility services. 

### Evidence-informed policy

Bastien Kolt et al. (124–126) argue for a shared view of research.

### Dementia prevention

Timothy Daly et al. (127–130) examine ways to mitigate inequality. 

### Births, deaths and demographic dividends 

David E Bloom et al. (134–136) explore implications of changes in population structure.

